# Biochemical Characterization and Disease Control Efficacy of *Pleurotus eryngii*-Derived Chitosan—An In Vivo Study against *Monilinia laxa*, the Causal Agent of Plum Brown Rot

**DOI:** 10.3390/plants13182598

**Published:** 2024-09-17

**Authors:** Ippolito Camele, Amira A. Mohamed, Amira A. Ibrahim, Hazem S. Elshafie

**Affiliations:** 1 Department of Agricultural, Forestry, Food and Environmental Sciences (DAFE), University of Basilicata, 85100 Potenza, Italy; 2 Department of Basic Science, Zagazig Higher Institute of Engineering and Technology, Zagazig 44519, Egypt; aa.adaim@science.zu.edu.eg; 3 Botany and Microbiology Department, Faculty of Science, Arish University, Al-Arish 45511, Egypt; amiranasreldeen@sci.aru.edu.eg

**Keywords:** biopesticides, natural products, antimicrobial activity, mushroom, plant diseases, post-harvest diseases

## Abstract

Chitin (Ct) is a crucial biopolymer present in fungi, algae, arthropods, and is usually obtained from crustacean shells. Chitosan (Cs) is a derivative from Ct deacetylation, and possesses numerous uses in various agro-industrial fields. Research on fungal-derived Ct and Cs is mostly focused on pharmaceutical uses, however their uses for plant disease control remain less explored. The main objective of the current study is to evaluate the possibility of using chitosan obtained from mushroom *Pleurotus eryngii* (Cs-Pe) for controlling some phytopathogens compared to commercial chitosan (C.Cs). This study is focused on the following key areas: (i) extracting Ct from *P. eryngii* mycelium and converting it to Cs through deacetylation, using both bleaching and non-bleaching methods; (ii) conducting a physico-chemical characterization and in vitro evaluation of the antimicrobial activity of the obtained Cs; (iii) performing an in vivo assessment of the phytotoxic and cytotoxic effects of Cs; and (iv) investigating in vivo the impact of the studied chitosan on fruit quality and its biocontrol efficacy against *Monilinia laxa* infections in plum fruits. Results showed that Cs-Pe, especially the unbleached one, displayed promising in vitro antimicrobial activity against the majority of tested pathogens. Regarding the cytotoxicity, the highest significant increase in cell abnormality percentage was observed in the case of C.Cs compared to Cs-Pe. In the in vivo study, Cs-Pe acted as a protective barrier, lowering and/or preventing moisture loss and firmness of treated plums. The studied Cs-Pe demonstrated notable efficacy against *M. laxa* which decreased the fruits’ percentage decline. These results strongly suggest that Cs derived from *P. eryngii* is a potential candidate for increasing plums’ shelf-life. This research shed light on the promising applications of *P. eryngii*-derived Cs in the agri-food field.

## 1. Introduction

**Figure 1:**
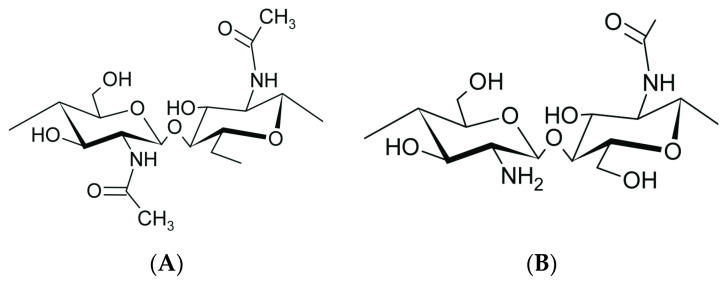
Chemical structure of chitin (**A**) and chitosan (**B**).

Chitin (Ct) is one of the most abundant biopolymers in nature [1,2], and it can be found in different organisms as a supportive and protective component of the exoskeleton of arthropods, fungal cell walls, and some algae [3]. Chitin is traditionally extracted from crustacean shells, squid skeletons, and the cuticles of insects [4,5,6]. Currently, commercial Ct and its deacetylated derivate, chitosan (Cs), are produced from shrimp and crab shells as by-products of the sea-food industry [1,2,7]. In addition, beside the textile and paper industries, Ct and Cs are also used in the food industry, agriculture, wastewater treatment, tissue engineering, biomedical, and biotechnological fields [8,9]. The chemical structures of Ct and Cs consist of 2-amino-2-deoxy-D-glucopyranose and 2-acetamide-2-deoxy-D-glucopyranose units, respectively, linked together by various amounts of glycosidic β-1,4 bonds (Figure 1A,B) [10]. The conversion from Ct to Cs involves the removal of the acetyl group (-CH_3_CO) from the acetyl glucosamine monomer within the Ct chain. This action releases the amino group (-NH_2_), converting it into a glucosamine monomer and ultimately yielding Cs (Figure S1). 

The traditional extraction of Ct from crustacean shells requires strong alkali, high temperature treatment, and has seasonal supply as well as geographical limitations [11,12]. Since the late 1970s, after White et al. [11] introduced a laboratory-scale method for extracting chitosan from *Mucor rouxii* mycelia, numerous protocols have been devised to use fungal biomass as an alternative source for chitosan production, instead of crustacean shells [11]. It is well-known that fungal cell walls are rich in neutral polysaccharides, glycoproteins with minor amounts of galactosamine polymers, polyuronides, melanin, lipids, and chitin which give rigidity to the cell wall [10,13,14,15]. The taxonomic groups Zygomycetes, Ascomycetes, Basidiomycetes, and Mitosporic fungi are known to contain Ct in their cell walls. In contrast, Oomycetes, which were previously classified as fungi, are characterized by the presence of cellulose instead of Ct [14]. Therefore, the extraction of Ct from fungi has attracted significant importance recently. Although insect-derived chitin/chitosan has properties similar to those from crustaceans, fungal Ct/Cs provides distinct advantages, including superior particle size uniformity and lower molecular weight, compared to that derived from both insects and crustaceans [16,17].

Some edible fungi have recently attracted attention for their nutritional and medicinal properties [18]. Additionally, mushrooms are significant sources of biologically active compounds, which have important effects on the immune system and have antimicrobial activities [19]. Among the most important edible fungi, genus Pleurotus has been extensively investigated for its biological properties due to the presence of some bioactive compounds such as polysaccharides, enzymes, and proteins [20,21,22,23]. In particular, *Pleurotus eryngii* (DC.) Quél., also known as the king oyster mushroom, is native to the Mediterranean basin of Europe and the Middle East [24], and known in Italy as “Cardoncello”. This fungus is rich in vitamins and minerals, and has a low content of carbohydrates, cholesterol, and other bioactive substances that can improve the immune response [25,26]. Several studies have explored the pharmaceutical applications of Ct derived from edible mushrooms, particularly the Pleurotus species [27]. However, to our knowledge, there has been no significant research investigating the potential use of Ct and/or Cs extracted from *P. eryngii* for plant disease control.

The main objectives of the current study are to: (i) physiochemically characterize the obtained Cs from *P. eryngii* (Cs-Pe); (ii) evaluate the in vitro antimicrobial activity of bleached and unbleached Cs-Pe against various phytopathogenic bacteria and fungi compared to commercial chitosan (C.Cs); (iii) assess the phytotoxic and cytotoxic effects of the studied Cs-Pe; and (iv) evaluate the in vivo effects of the studied Cs-Pe on plums’ quality and their potential biocontrol activity against *Monilinia laxa*, the causal agent of brown-rot disease. 

## 2. Results

### 2.1. Biomass Quantification

**Figure 2:**
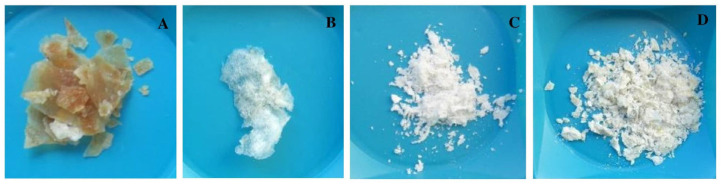
Chitin extracted from *P. eryngii* and deacetylated chitosan. (**A**) U.Ct.Pe; (**B**) B.Ct.Pe; (**C**) U.Cs.Pe; (**D**) B.Cs.Pe. Photos were taken by a Nikon Digital Camera (D5100) (Nikon Imaging Japan Inc., Tokyo, Japan).

Table 1 summarizes the key findings, including the weight of produced fungal biomass in three different nutrient media: (i) potato dextrose broth supplemented with 50 g of wheat powder (PDBW); (ii) malt extract broth (MEB); and (iii) nutrient broth (NB). Table 1 also illustrates the dry weight and yield percentage of extracted Ct and deacetylated Cs, both bleached and unbleached. In particular, the highest significant biomass production was achieved with PDBW, with 65.5 g of fresh weight (F.Wt) and 31.6 g of dry weight (DW). MEB produced 12.6 g of FW and 4.4 g of DW, while NB produced 22.3 g of FW and 9.4 g of DW of biomass. On the other hand, the produced biomass from PDBW was used for the extraction of chitin and the deacetylation process to obtain the chitosan. The unbleached chitin (U.Ct.Pe) and bleached chitin (B.Ct.Pe) yielded 13.3 g DW (42.1% relative to fungal biomass) and 2.6 g (8.2% relative to fungal biomass), respectively. In contrast, unbleached chitosan (U.Cs.Pe) and bleached chitosan (B.Cs.Pe) produced 6.4 g DW (48.1% relative to Ct) and 1.9 g (73.1% relative to Ct), respectively (Figure 2).

### 2.2. Physicochemical Characteristics of Chitin and Chitosan

#### 2.2.1. FT-IR Analysis

**Figure 3:**
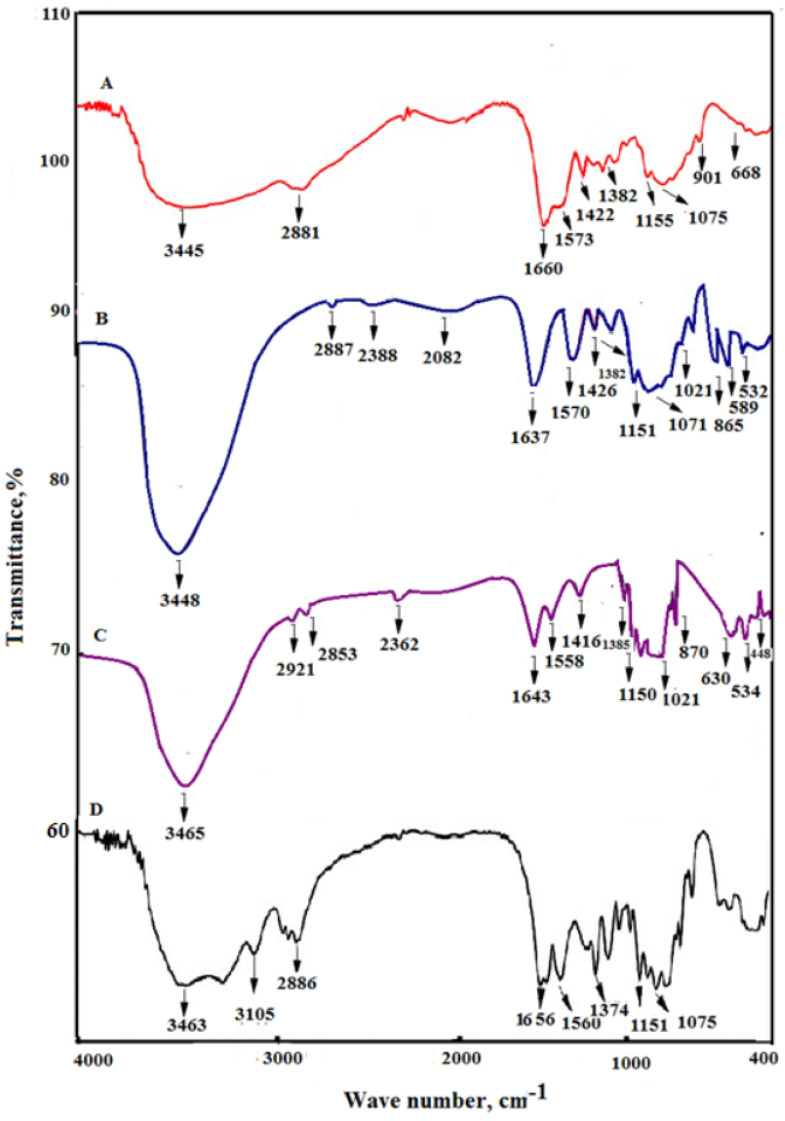
FT-IR spectrum of C.Cs (A), B.Cs.Pe (B), U.Cs.Pe (C), and Ct (D).

The study’s findings, which are shown in Figure 3, showed the existence of many absorption bands in the 4000–400 cm^−1^ region. The FT-IR spectrum is of B.Cs.Pe, U.Cs.Pe, C.Cs, and Ct. After the depolymerization process, the Cs structure stays stable, as evidenced by the IR spectrum, which was quite close to native Cs [28,29]. Table 2 illustrates the FT-IR spectra of studied Cs.Pe compared to C.Cs and Ct. In particular, a band cantered around 3450 cm^−1^ signifies the existence of an OH group [30,31]. The band around 2887 cm^−1^ is attributed to C-H stretching. In addition, the presence of the C=O group is shown by a band at around 1649 cm^−1^. The intensity of this peak was decreased and a shift occurred compared to C.Cs due to the decrease for the acetyl group in B.Cs.Pe and U.Cs.Pe [32]. The presence of a band around 1570 cm^−1^ corresponds to the N-H bending group in the B.Cs.Pe and shifted at 1558, 1573, and 1560 cm^−1^ at U.Cs.Pe, C.Cs, and Ct, respectively. The bands around 1150 cm^−1^ and 1075 cm^−1^ were attributed to C-C and C-N groups, respectively [33,34]. Compared to the FT-IR spectra of Ct, there was a decrease in the shift and also intensity peak of C=O in the case of the extracted Cs due to the decrease of acetyl content because of the deacetylation process performed in Ct.

#### 2.2.2. Molecular Weight and Viscosity

The viscosity of B.Cs.Pe and U.Cs.Pe resulted in 1.21 and 1.02 centipoise (cP), whereas, their molecular weights (M.Wt) were equal to 20.5 KDa and 17.3 KDa, respectively. In fact, high M.Wt Cs had a higher viscosity than a low M.Wt one. Omogbai and Ikenebomeh [35] reported that the viscosity of Cs is an important factor which determines its applications and significantly affects its antimicrobial activities.

#### 2.2.3. Degree of Deacetylation

The degree of deacetylation (DD%) in Ct and Cs, which is essential for determining their physicochemical properties, can be analyzed using infrared spectroscopy. Infrared spectra of Ct and Cs from *P. eryngii* and C.Cs were used to measure the DD%, which was found to be 72.47, 81.12, 80.01, and 62.57% for B.Cs.Pe, U.Cs.Pe, C.Cs, and Ct, respectively. 

### 2.3. Antimicrobial Activity

Antibacterial. The results of the antibacterial assay demonstrated the potent efficacy of U.Cs.Pe and B.Cs.Pe against all tested pathogenic bacteria (Figure S2). Notably, the U.Cs.Pe exhibited the lowest minimum inhibitory concentration (MIC) value (0.375 mg/mL) for *Clavibacter michiganensis* compared to B.Cs.Pe (0.75 mg/mL) and C.Cs (1.5 mg/mL). Regarding *Escherichia coli*, the lowest MIC value (1.5 mg/mL) was observed in the case of U.Cs.Pe and B.Cs.Pe, compared to C.Cs (3.0 mg/mL). Regarding *Xanthomonas campestris*, the lowest MIC value (1.5 mg/mL) was observed in the case of U.Cs.Pe, compared to both B.Cs.Pe and C.Cs (3.0 mg/mL) (Table 3). The positive control showed MIC values ranged between 50 µg/mL in the case of *C. michiganensis* and *P. fluorescence*, and 100 µg/mL in the case of *E. coli* and *X. campestris*.

Concerning the bactericidal results, the minimum bactericidal concentration (MBC) of U.Cs.Pe were 6.0, 3.0, 0.75, and 3.0 mg/mL for *E. coli*, *X. campestris*, *C. michiganensis*, and *Pseudomonas fluorescence*, respectively. In comparison, B.Cs.Pe exhibited MBC values of 6.0, 6.0, 1.5, and >6.0 mg/mL against the same bacteria (Table 4).

Antifungal. The results of the antifungal assay demonstrated that the studied Cs has a potent efficacy against all tested pathogenic fungi (Figure S3). In particular, the lowest MIC values exhibited by the U.Cs.Pe were 1.5, 0.375, and 0.75 mg/mL compared to B.Cs.Pe ≥3.0, 1.5, and 1.5 mg/mL against *Penicillium expansum*, *Botrytis cinerea*, and *Monilinia laxa*, respectively (Table 5). Whereas, the MIC values for C.Cs displayed 1.5 mg/mL against all tested fungi. The positive control showed a MIC value equal to 100 µg/mL for all tested fungi.

Concerning the fungicidal results, the minimum fungicidal concentration (MFC) of U.Cs.Pe demonstrated lower values of 6.0, 3.0, and 0.75 mg/mL compared to 6.0, 6.0, and 1.5 mg/mL in the case of both B.Cs.Pe and C.Cs, against *P. expansum*, *B. cinerea*, and *M. laxa*, respectively (Table 6).

### 2.4. Phytotoxicity

The phytotoxic effects of the studied chitosan were evaluated against three sensitive plants (*Lepidium sativum*, *Lactuca sativa*, and *Solanum lycopersicum*) at concentrations of 3000, 1500, and 750 ppm. The results are presented in Table 7. Specifically, for *L. sativum*, C.Cs exhibited the highest significant germination index (G.I.) of 53.4% at the lowest tested concentration of 750 ppm, compared to other tested Cs. For *L. sativa*, U.Cs.Pe demonstrated the highest significant G.I. of 50.6% at 750 ppm, which is very close to the control. In the case of *S. lycopersicum*, both B.Cs.Pe and U.Cs.Pe showed the highest significant G.I. (63.5%) and (54.4%), respectively, which closely match the control. All tested Cs exhibited very low significant G.I. at the highest concentration of 3000 ppm. The notable phytotoxic activity of the Cs extracted from *P. eryngii* suggests its potential use as a natural herbicidal agent against harmful weeds.

### 2.5. Cytotoxicity

The cytotoxic effect of the C.Cs and Cs.Pe on the mitotic index (MI), phase index (PI), and total abnormalities (Tab) of *Vicia faba* root tips was illustrated in Table 8. The highest significant MI was found in the case of U.Cs.Pe (16.92%) followed by B.Cs.Pe (16.87%), while the lowest MI was recorded in the case of C.Cs (12.67%) compared to control cells (14.87%). 

The PI included mitotic index and abnormality percentages for each cell division stage, except the interphase, as illustrated in Table 8. In particular, the highest MI at the prophase stage was 15.98%, presented in C.Cs, whereas the lowest MI at the prophase was found in U.Cs.Pe with a value of 13.88%, compared to the control (16.98%). The highest MI % at the metaphase was 57.87% for C.Cs, while the highest values at the anaphase (23.87%) and telophase (22.02%) were recorded in U.Cs.Pe. The highest significant values in abnormality percentages at the prophase, metaphase, anaphase, and telophase stages were observed in the case of C.Cs, with values of 3.87, 23.67, 7.45, and 3.54%, respectively. 

The Tab % of mitosis was found in C.Cs (38.53%), while the lowest values were 17.08 and 18.65% in the case of B.Cs.Pe and U.Cs.Pe, respectively, compared to the control (24.64%). The Tab % at the interphase was presented in C.Cs with a value of 1.87%, compared to the control 0.87%, whereas there were no any abnormalities at the interphase stage for other treatments. 

**Figure 4:**
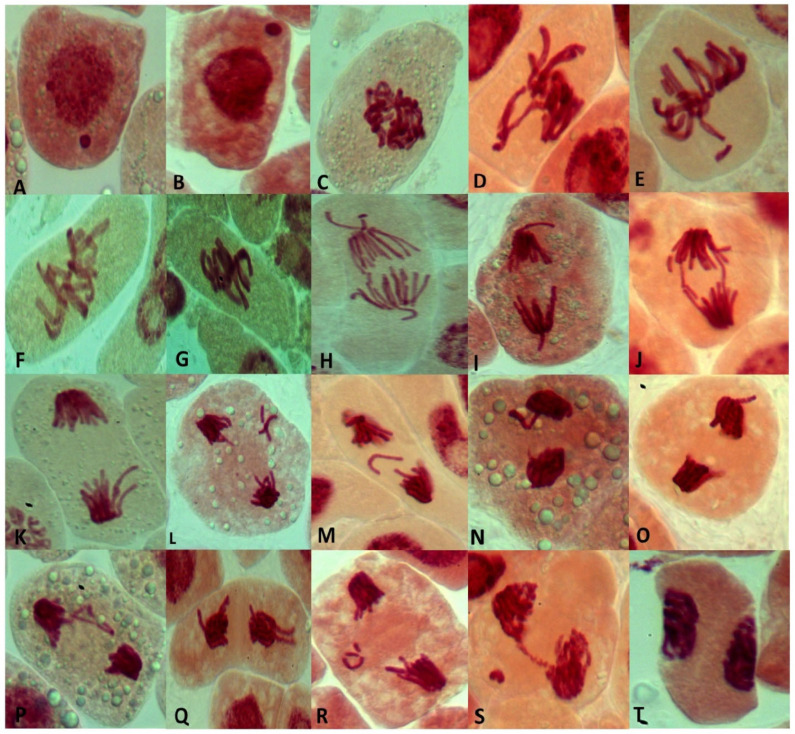
Types of mitotic abnormality induced by treatment of *Vicia faba* root tips by different tested Cs. (**A**,**B**): micronucleus at interphase; (**C**): stickiness at metaphase; (**D**): disturbed metaphase; (**E**): non-congression at metaphase; (**F**,**G**): oblique at metaphase; (**H**,**I**): disturbed anaphase; (**J**): bridges at anaphase; (**K**): late separation at anaphase; (**L**,**M**): laggard at anaphase; (**N**,**O**): disturbed at telophase; (**P**,**Q**): late separation at telophase; (**R**): laggard at telophase; (**S**): bridge at telophase; and (**T**): diagonal telophase (X = 100).

Different types of chromosomal abnormality at different stages of *V. faba* root tip cell division were recorded in Figure 4. The micronucleus at the interphase was found in C.Cs (Figure 4A,B). At the metaphase stage, the common abnormalities were presented as stickiness (Figure 4C), disturbed (Figure 4D), non-congression (Figure 4E), and oblique (Figure 4F,G). At the anaphase, they were disturbed (Figure 4H,I), bridges (Figure 4J), late separation (Figure 4K), and laggard (Figure 4L,M). At the telophase, the common types of abnormality were disturbed (Figure 4N,O), late separation (Figure 4P,Q), laggard (Figure 4R), bridge (Figure 4S), and diagonal telophase (Figure 4T). 

### 2.6. In Vivo Fruit-Coating Assay

#### 2.6.1. Quality Parameters

The selected U.Cs.Pe showed a strong ability to form a protective coating-layer on fruit surfaces. On the other hand, there was not significant effect on the quality parameters of control fruitss treated with 1% acetic acid and/or glycerol.

(I) Weight loss

**Figure 5:**
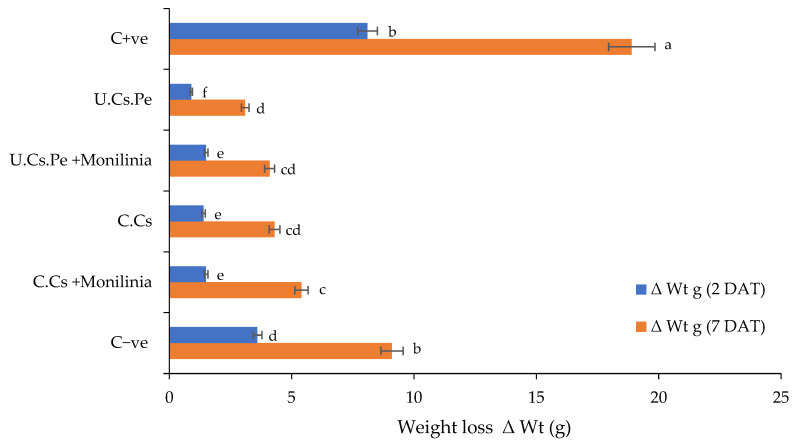
Weight loss of plums after coating treatment. Where: C + ve: control positive (fruits infected only with *M. laxa*); AA: acetic acid at 1%; Gly: glycerol. Bars with different letters for each period are significantly different at *p* < 0.05. Data for each bar are expressed as the mean of three replicates ± SDs.

**Figure 6:**
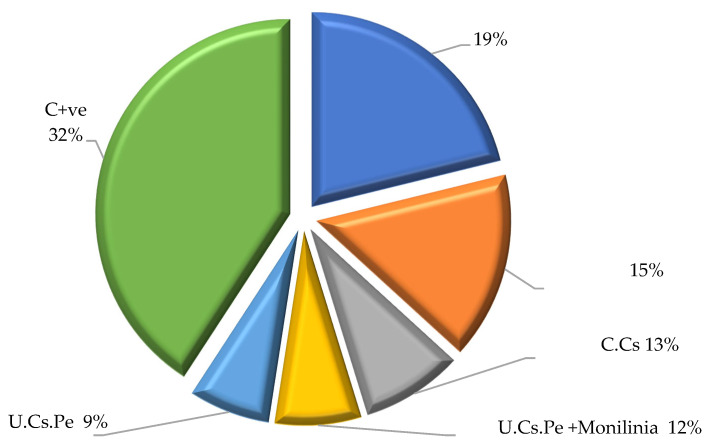
Weight loss percentage of plums at the end of conservation period.

The influence of different coatings on weight loss percentages is depicted in Figure 5. In particular, the highest significant weight loss (27%) was observed in the case of the positive control (C + ve), i.e., fruits infected with *M. laxa* (Figure 6), whereas, the lowest weight loss (4%) was observed in the case of fruits treated with U.Cs.Pe, followed by fruits treated with U.Cs.Pe and infected with *M. laxa* (7%) (Figure 6).

(II) Peel color change

**Figure 7:**
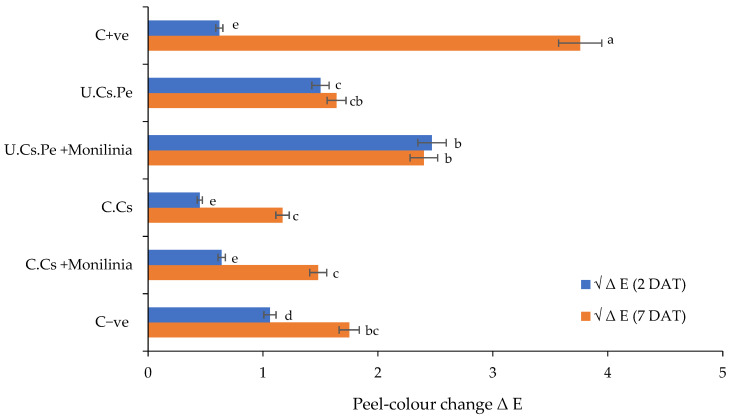
Peel color change in plums after coating treatment. Bars with different letters for each period are significantly different at *p* < 0.05 using the Tukey B test. Data for each bar are expressed as the mean of three replicates ± SDs.

The obtained results of the influence of different coatings on peel color are illustrated in Figure 7. The treatment with U.Cs.Pe did not show significant variation in peel-color, even 7 DAT (Figure S4). In particular, fruits treated with U.Cs.Pe, whether infected or not, exhibited moderate changes in peel color with ΔE values ranging between 1.5 and 2.4 relative to the negative control (C − ve) (7 DAT). On the other hand, the highest significant change in peel color was observed in the case of C + ve, with a Δ E 3.76 (7 DAT). In addition, C.Cs-treated fruits showed the least change in peel color with a ΔE value of 1.17.

(III) Firmness

**Figure 8:**
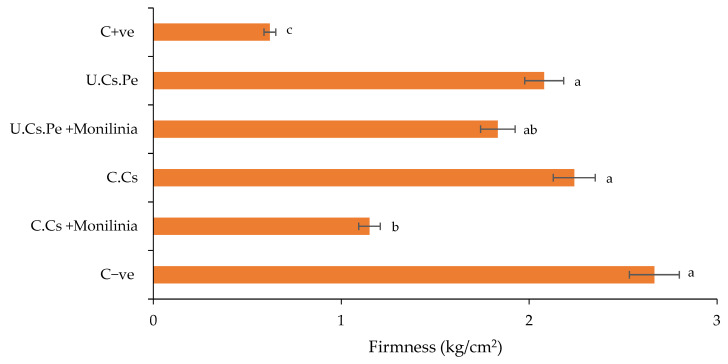
The firmness of plums after coating treatment at the end of the conservation period. Bars with different letters are significantly different at *p* < 0.05. Data for each bar are expressed as the mean of three replicates ± SDs.

The results of the studied coating treatments on fruit firmness 7 DAT are illustrated in Figure 8. Firmness is a crucial visual quality parameter for fresh market fruits as it significantly influences post-harvest quality and shelf life. Treated fruits with U.Cs.Pe showed a low, insignificant reduction in firmness compared to C − ve and those treated with C.Cs. Notably, infected fruits treated with U.Cs.Pe exhibited only a slight significant decrease in firmness, with a value of 1.83 kg/cm^2^, compared to those infected and treated with C.Cs, which showed a significant firmness reduction to 1.15 kg/cm^2^.

(IV) Sugar content

**Figure 9:**
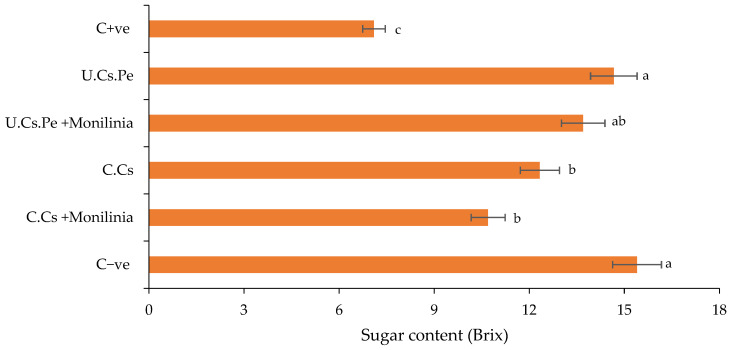
The sugar content of plums after coating treatment at the end of the conservation period. Bars with different letters are significantly different at *p* < 0.05. Data for each bar are expressed as the mean of three replicates ± SDs.

Specifically, the results from treated fruits with U.Cs.Pe, either infected or not, showed a slight decrease in sugar content even at 7 DAT (13.70 and 14.67 Brix, respectively), compared to untreated controls (15.40 Brix) (Figure 9), whereas, fruits treated with C.Cs, whether infected or not, exhibited sugar contents of 10.70 and 12.33 Brix, respectively.

#### 2.6.2. Decay Evaluation

**Figure 10:**
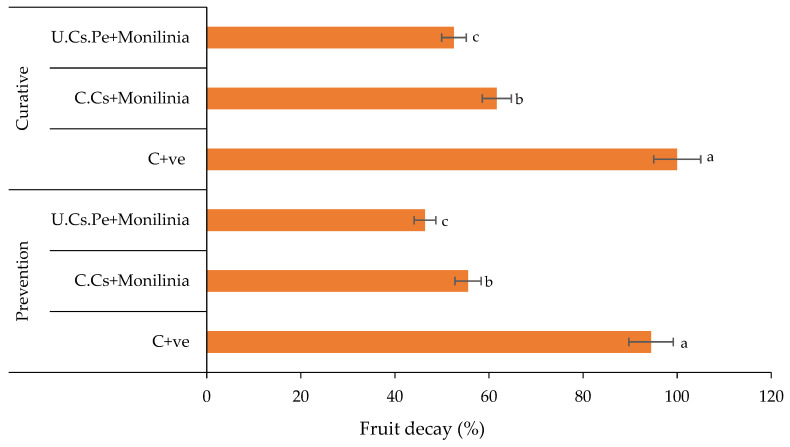
The decay percentage of plums after coating treatment and artificial infection with *M. laxa*. Bars with different letters for each method are significantly different at *p* < 0.05. Data are expressed as the mean of three replicates ± SDs.

After 5 days of storage, untreated fruits, or those treated with C.Cs and infected with *M. laxa*, exhibited brown-rot symptoms. In contrast, fruits treated with U.Cs.Pe showed a reduction in disease symptoms, with decay percentages of 46.4 and 52.6% for preventive and curative treatments, respectively (Figure 10). The C + ve showed the highest decay percentage ranged between 94.4 and 100% with regard to prevention and curative treatment, respectively, whereas, C.Cs showed a moderate decay percentage of 55.6 and 61.7% in the case of prevention or curative treatment, respectively (Figure S5). On the other hand, the decay percentage of acetic acid (1%), either for prevention or curative treatments, were 61.1 and 77.8%, respectively, excluding any effect on the Cs efficacy.

## 3. Discussion

In our study, Ct was extracted from the cell wall of *P. eryngii,* and subsequently underwent a deacetylation process to obtain Cs, which is soluble in acidic solutions. Chitin’s insolubility in water and many organic solvents is due to its primary structure, comprising nanofibrils encapsulated within a protein matrix [36]. These nanofibrils, typically forming crystallites approximately 3 nm in diameter, are stabilized by hydrogen bonds between the amine and carbonyl groups, as reported by Ibrahim et al. [37].

The M.Wt and viscosity of the Cs obtained in our study were similar to the findings of Pochanavanich and Suntornsuck [38]. They reported that the viscosity of Cs extracted from *Aspergillus niger*, *Rhizopus oryzae*, *Lentinus edodes*, and *P. sajo-caju* ranged between 3.1 and 6.2 cP, corresponding to a M.Wt between 27 and 190 KDa. This is significantly lower than the viscosity of commercial crab shell Cs, which is 372.7 cP. Furthermore, our results closely match those of Khalaf [39], who found that the viscosity of fungal Cs extracted from *A. niger*, *P. citrinum*, *Fusarium oxysporum*, and *R. oryzae* ranged between 2.7 and 6.8 cP, corresponding to a M.Wt between 45.54 and 113.71 KDa. On other hand, our results are slightly higher than the results obtained by Ugochukwu et al. [40], who reported that the extracted Cs from *P. ostreatus* exhibited viscosity ranging between 0.38 and 0.37 cP, corresponding to M.Wt ranging between 6.48 and 6.25 KDa. It is important to emphasize that low molecular weight (LMw) Cs has a high charge density and excellent solubility, making it highly effective and widely applicable in the pharmaceutical, biomedical, and food industries [41]. Additionally, LMw Cs is characterized by high permeability due to its low viscosity, a property that is crucial for biological applications and enhances its antimicrobial activity [42]. Many previous studies have reported that Ct and Cs extracted from insects showed, generally, properties similar to those extracted from crustaceans [15,17], whereas, the Ct/Cs extracted from fungi are characterized by higher particle size uniformity and LMw, compared to those extracted from insect and crustaceans [16]. In addition, the Ct/Cs derived from shellfish exhibited evenly distributed acetyl groups, whereas fungal Cs tended to exhibit a clustering pattern in its acetylation [17]. It is important to highlight that chitosan, with a low molecular weight, has been reported to reduce the tensile strength and elongation of chitosan membranes, while enhancing their permeability, as reported by Rong and Horng [42]. 

On the other hand, the results of the degree of acetylation were similar to those reported by Pochanavanich and Suntornsuk [38] who reported that the Cs produced from different fungal genera, among them, *Aspergillus*, *Rhizopus*, *Pleurotus*, etc., had a degree of deacetylation ranging between 84 and 90%. The DD% is linked to the molecule’s positive charge density, enhancing its bioactivity in various applications such as antimicrobial and coagulation agents, as reported by Khalaf [39].

The significant antimicrobial activity of the studied Cs, especially U.Cs.Pe, may be attributed to its ready solubility in organic solvents, enhanced by the presence of free amino groups. These amino groups are responsible for conferring the polymer’s basic behaviour and cationic properties, thereby increasing its reactivity [43]. In addition, the presence of amino and hydroxyl groups in the polymer structure of Cs allows them to penetrate the microbial cell membranes, rupturing their integrity and causing cell death [43]. This characteristic, in turn, raises Cs solubility in water and subsequently improves its adhesion to the negatively charged microbial cell wall surfaces [44]. On the other hand, numerous studies have reported that the antimicrobial activity of Cs is closely related to its M.Wt. Specifically, Cs with different M.Wt has been observed to exhibit varying levels of effectiveness against different species of bacteria [45]. Some studies indicate that increasing the M.Wt of Cs is associated with reduced activity, whereas other research suggests that Cs with a high Mw exhibits greater activity compared to chitosan with a low Mw [46]. Hosseinnejad and Jafari [47] reported that the components of Cs are absorbed into the surface of microbial cells, where they are thought to exert antimicrobial activity. This interaction makes the lipid cell membrane more permeable, allowing essential substances to leak out of the cell, ultimately leading to cell death. However, the relationship between Cs’s molecular weight and its antimicrobial activity can vary depending on specific conditions and the pathogen under study. Further research is required to fully elucidate this relationship and optimize the use of chitosan for antimicrobial applications.

Regarding the in vivo trial, the notable efficacy of U.Cs.Pe was evident in its ability to form a protective coating layer on the surface of fruits. This coating can alter gas permeation, lower the respiration rate, and reduce water loss, leading to better weight maintenance and enhanced firmness retention in the fruits [48,49]. In particular, the monitored quality parameters, such as weight loss, has a notable impact on the perceived flavour and freshness of fruits, as reported by Ribeiro and de Freitas [50]. In particular, the significant weight loss observed during storage is primarily due to increased water loss through evaporation and transpiration. This water loss reduces the turgor pressure within the fruits’ cells, leading to tissue shrinkage and overall weight reduction [51]. Our findings revealed that fruits treated with Cs.Pe exhibited minimal weight loss percentages in agreement with the findings of Li et al. [52], who studied the effect of coating treatment with Cs on the quality parameters of plums during storage. Their conclusion highlighted that the Cs treatment acted as a physical barrier, effectively inhibiting moisture loss and reducing water flow from the fruits’ tissues, and consequently delayed the shrinkage and dehydration of the plums [52]. The low reduction in fruits’ firmness after treatment with Cs.Pe could be due to the coated film which protects the fruits from the transpiration rate, and hence delays the over-softening of plums, as reported by Zhang et al. [53].

On the other hand, our results showed that treatment with Cs.Pe had no effect on the sugar content of treated fruits during storage. In another study conducted by Kambhampati and Kotra [54], it was reported that there was a rapid decline in the titratable acidity of kiwifruit samples during storage, compared to the Cs-coated, fresh-cut kiwifruit samples. In the latter study, the authors explained also that the Cs-coating treatment could make an effective film and reduce metabolic activities which inhibit the metabolic rate of the titratable acids, maintaining higher acidity during post-harvest. Consequently, the results of in vivo trials suggested that Cs derived from natural sources, such *P. eryngii*, might be used to extend the shelf life of fruits.

The results obtained from the application of Cs.Pe to control fungal disease demonstrated a promising effect in lowering the percentage of plums’ decay caused by *M. laxa* infection. A related study by Brulé et al. [55] reported that Cs treatment is an efficient way to manage plant diseases either pre- or post-harvest, including various fungal diseases that affect grapevines, like powdery mildew, grey mould, and downy mildew. On the other hand, Zeng et al. [56] reported that the treatment of navel oranges with 2% Cs can significantly reduce the disease incidence and lesion diameter caused by *P. italicum* and *P. digitatum*, inducing disease resistance by regulating H_2_O_2_ levels, antioxidant enzymes, and the ascorbate–glutathione cycle.

The beneficial biological effects of Cs have been reported in various agricultural applications. For instance, Cs used in seed-coating technology can trigger an innate immune response in developing roots, effectively targeting parasitic cyst nematodes while preserving beneficial organisms [57]. Moreover, Cs can serve as a natural seed treatment and plant growth promoter, acting as an eco-friendly biopesticide that enhances plants’ innate defence mechanisms against fungal infections. This approach reduces reliance on synthetic fungicides and contributes to environmental protection. 

Fungal-derived chitosan presents several advantages over crustacean-derived chitosan in agriculture. Notably, fungal chitosan is more sustainable because it can be produced continuously through bio-fermentation, making it a more renewable resource. Additionally, chitin and chitosan extracted from fungi is characterized by its greater particle size uniformity and lower molecular weight compared to those extracted from crustaceans, which enhances their solubility and, consequently, their bioactivity.

## 4. Materials and Methods

### 4.1. Tested Fungi

The tested *P. eryngii* (collection number 1544), stored at 4 °C in the fungal collection of the Department of Agricultural, Forestry, Food and Environmental Sciences (DAFE), University of Basilicata, Potenza (Italy) was sub-cultured on Potato Dextrose Agar (PDA) nutrient media for 96 h at 24 °C. 

### 4.2. Biomass Quantification

To determine the most effective nutrient medium for maximizing biomass production, three different nutrient media were prepared: (i) PDBW; (ii) MEB; and (iii) NB. Three Erlenmeyer flasks (1 L) containing 800 mL for each tested media were prepared, autoclaved, inoculated with two agar pieces (Ø 0.5 cm^2^) of fresh fungal mycelium, and incubated in agitation (180 rpm) for 10 days at 24 °C in a rotary-incubator (Heidolph WB 2000, Labexchange, Germany) [58]. The incubated broths were collected and centrifuged at 20,000× *g* for 15 min. The biomasses were harvested, washed twice with sterile distilled water (SDW), and dried under laminar flow overnight. The biomass weights were measured following the dried-weight method as reported by Álvarez et al. [59], and the highest biomass quantity was chosen for subsequent analysis.

### 4.3. Chitin Extraction and Deacetylation

The Ct extraction was carried out as described by Hassainia et al. [60]. In particular, the extraction method of Ct and transformation to Cs were performed through two main steps: (i) deproteinization of fungal biomass; (ii) heterogeneous deacetylation of Ct. 

Deproteinization. Fungal biomass was dried in an oven at 60 °C for 48 h, and then ground into powder using liquid nitrogen. The biomass was demineralized using 0.5 M formic acid (CH_2_O_2_) for 1 h at room temperature while stirring. Subsequently, the sample was washed with SDW to restore a neutral pH (7.0). The demineralized biomass was then stirred with 2 M NaOH for 2 h at 80 °C to remove proteins. Following deproteinization, half of the Ct sample underwent a bleaching procedure with 5% hydrogen peroxide (H_2_O_2_) for 1 h at 90 °C, while the other half remained untreated as unbleached. The bleaching process of Ct removes pigments, proteins, and impurities, resulting in a purer product. This enhances its color, material properties, biocompatibility, and functionality, making it suitable for biomedical, pharmaceutical, food, and cosmetic applications. Thus, bleaching ensures Ct meets the necessary standards for these uses.

Heterogeneous deacetylation. The Cs was obtained by heterogeneous deacetylation of both bleached and unbleached Ct [61]. As briefly reported: 50 mL of 12 M NaOH were added to 40–80 mg of dried Ct and incubated for 2 h at 100 °C. Flakes of Cs were obtained by thoroughly rinsing the solid residue with SDW. The collected Cs was dissolved in 1% acetic acid (CH₃COOH) at 40 °C and the pH was adjusted to 7.0. Acetic acid (1%) is used to protonate the amino groups, converting them into ammonium ions, which enhances the solubility of Cs. 

The yield percentages of U.Ct.Pe, B.Ct.Pe, U.Cs.Pe, and B.Cs-Pe were assessed according to the following Equation (1):

(1) $$\mathrm{Yield}\left(\%\right)=\frac{\mathrm{Dry}\mathrm{weight}\mathrm{of}\mathrm{chitin}\mathrm{or}\mathrm{chitosan}\left(g\right)}{\mathrm{Dry}\mathrm{weight}\mathrm{of}\mathrm{fungal}\mathrm{biomass}\left(g\right)}\times 100$$

### 4.4. Physicochemical Characterization of Chitin and Chitosan

#### 4.4.1. Fourier Transform Infrared Spectroscopy (FT-IR) Analysis

The FT-IR analysis was applied to samples with better crystallinity to confirm the presence of functional groups of extracted Ct and Cs. The studied Ct and Cs samples were mixed with potassium bromide (KBr) in the range from 4000–400 cm^−1^, and a Shimadzu FT-IR 460 (Shimadzu 8400S, Tokyo, Japan) apparatus was used to record the spectra.

#### 4.4.2. Determination of Molecular Weight and Viscosity

Molecular weight is one of the most fundamental parameters in characterizing polymers and determining their activity. The M.Wt of Cs-Pe was determined by measuring its viscosity using the Mark–Houwink–Sakurada (MHS) Equation (2) because the viscosity of a polymer is directly related to its M.Wt [62,63].

M.Wta = K/η(2)

where: (M.Wt) molecular weight; (η) intrinsic viscosity; (K) and (a) constants (K = 0.078; a = 0.76) for given solute–solvent system and temperature.

The determination of constants (K) and (a) from the intrinsic viscosity data requires either a series of monodisperse polymers of known M.Wt or a series of polydisperse polymer samples with known viscosity-average M.Wts. The intrinsic viscosity of Cs-Pe has been determined experimentally by capillary viscometry using an Ostwald viscometer (Model AMV-200, Paar Physica Edison, NJ, USA). The method is based on the measurement of the flow (t0) of the same volume of solvent (1% acetic acid) or Cs solution (t) contained between the two points (lines) marked on the viscometer using a chronometer with an inclination angle of 15 degrees and a capillary diameter of 0.9 mm. These parameters were chosen in order to minimize adjustments for kinetic energy and shear, combined with the usage of solution concentrations of less than 1% (*w*/*v*) [62,63]. The viscosity (η) has been calculated following Equation (3).

η = (t − t0)∕t0(3)

where: (η) intrinsic viscosity; (t0) the flow time of 1% acetic acid; (t) the flow time of chitosan solution contained between the two points (lines).

#### 4.4.3. Determination of Degree of Deacetylation (DD %)

According to Stamford [64], vibrational spectra in the infrared region was used to determine the degree of chitosan deacetylation by the ratio between the absorbance at wavelengths 1655 and 3450 cm^−1^, and the DD % was calculated following Equation (4), as proposed by Domzy and Roberts [65]:

DD (%) = 100 − [(A1655/A3450) × 115](4)

where: A1655 is the absorbance of wavelength 1655 cm^−1^, and A3450 is the absorption band at wavelength 3450 cm^−1^. The number “115” is the value of A1655/A3450 found in pure chitosan.

### 4.5. Antimicrobial Activity Assay

Tested bacteria. The tested bacteria were *E. coli* Migula (strain number ITM 103), *X. campestris* (Pammel) Dowson (strain number NCPPB 3035), *C. michiganensis* Smith (strain number C3718), and *P. fluorescens* (Flügge) Migula (strain number NCPPB 1796). 

Tested fungi. The tested fungi were *P. expansum* Link (collection number 1152 isolated from orange), *B. cinerea* Pers. (collection number 1931 isolated from strawberry), and *M. laxa* (Aderh. and Ruhland) Honey (collection number 1518 isolated from plum). All tested fungi were conserved in a refrigerator at 4 °C and recultivated on PDA media at 24 °C for 96 h.

All tested bacterial and fungal strains were conserved in the collection located in the Department of Agricultural, Forestry, Food and Environmental Sciences (DAFE), University of Basilicata, Potenza (Italy), and recultivated on King’s B (KB) medium [66] for bacteria, or PDA for fungi.

The MICs of studied U.Cs.Pe and B.Cs.Pe samples were carried out against all the above-tested phytopathogens, compared to C.Cs, using a 96-well microplate (Nunc MaxiSorp^®^, Vedbaek, Denmark) following the micro-dilution method [67]. Commercial chitosan derived from crustacean shells with molecular weight 100,000–300,000 Da (Thermo Scientific, Fair Lawn, NJ, USA) was used as a control. 

Stock solutions of U.Cs.Pe, B.Cs.Pe, and C.Cs at 6.0 mg/mL were dissolved in acetic acid (1%). The prepared solutions were stirred, filtered (0.22 μm), and conserved in a refrigerator at 4 °C. Six tested concentrations, labeled C1 to C6 (6.0, 3.0, 1.5, 0.75, 0.375, and 0.187 mg/mL), were prepared in SDW. One hundred microliters (µL) of each prepared concentration were added to the microplate wells, which had previously been supplemented with 50 µL of microbial suspension per well. The bacterial suspensions were adjusted to 10^8^ colony-forming units (CFU)/mL, while the fungal suspensions were adjusted to 10^6^ spores/mL, based on optical density measurements at 590 nm. All plates were incubated at 37 °C for 24 h in the case of bacteria, and 24 °C for 48 h in the case of fungi. The absorbance was measured using a microplate reader instrument (DAS s.r.l., Rome, Italy) at λ = 540 nm. Wells only filled with broth KB or PDB supplemented with acetic acid (1%) were considered as a control negative (C − ve). Two positive controls, Tetracycline (50 and 100 μg.mL^−1^) and Cycloheximide (100 and 500 μg.mL^−1^), for bacteria and fungi, respectively, were utilized mainly to ensure that the assay was carried out correctly, and also to validate the obtained results from different experimental samples. 

The MICs values for each tested pathogen were determined by monitoring the lowest tested concentration, which caused a significant reduction in microbial growth (values close to negative control). To determine the eventual bactericidal/fungicidal or bacteriostatic/fungistatic effects of each tested dose, the MBC and MFC were identified by re-culturing the tested micro-organisms from the lowest dose of each treatment that showed no visible growth. The MBC and MFC were determined to differentiate between the ability to completely kill the micro-organisms (bactericidal/fungicidal) or just inhibit the growth (bacteriostatic/fungistatic).

### 4.6. Phytotoxicity Assay

A bioassay based on seed germination and radical elongation (SG-RE) was carried out to evaluate the eventual phytotoxic effect of U.Cs.Pe, B.Cs.Pe, and C.Cs on *L. sativum* L. (garden cress), *S. lycopersicum* L. (tomatoes), and *L. sativa* L. (lettuce) [68]. The seeds were sterilized in 3% hydrogen peroxide for 1 min, rinsed twice with deionized SDW, and then immersed in each studied sample at 3.0, 1.5, and 0.75 mg/mL under shaking conditions (180 rpm/60 min). Seeds immersed only in SDW were considered as C − ve. Ten seeds for each tested plant species were transferred into glass Petri dishes (Ø 180 mm) containing two sterile filter papers (Whatman No. 1), pre-moistened with 2 mL of SDW or each single treatment, and then sealed with Parafilm. All petri dishes were incubated in a growth chamber at 30 °C with 85% relative humidity with 12-h photoperiod for 5 days. The experiment was carried out in triplicates. The seed germination (%) and the radical elongation (cm) were measured. The G.I. was calculated following Equation (5).

G.I. (%) = [(S.Gt × R.Et)∕(S.Gc × R.Ec)] × 100(5)

where: G.I. is the germination index; SG_t_ is the average number of germinated and treated seeds; RE_t_ is the average radical elongation of treated seeds; SG_c_ is the average number of germinated seeds of negative control; RE_c_ is the average radical elongation of negative control. 

### 4.7. Cytotoxicity Assay

The cytotoxicity of the studied Cs was assessed using a chromosome aberrations assay as follows: root tips of *Vicia faba* L. (broad bean) seeds cv. Skha1 were exposed to different Cs samples for 24 h. The root tips were then immersed in Carnoy’s solution (glacial acetic acid and ethanol, 1:3 *v*/*v*) and chilled for 48 h. The samples were then soaked for 5 min in SDW, followed by a hydrolysis in 1N HCl at 60 °C for 8 min. The root tips were rinsed with SDW, dyed with aceto-orcein for 4 h, treated with a drop of 45% acetic acid, and used for microscopic examination. The eventual chromosomal aberrations observed in the treated *V. faba* root tips are illustrated in the diagrammatic scheme (Figure S6).

### 4.8. In Vivo Fruit Treatment Assay

#### 4.8.1. Chitosan Treatment

The Cs solution was prepared by dissolving the studied chitosan sample, which showed a higher efficacy in vitro trial, in acetic acid (1%) at 6 mg/mL. Glycerol was utilized as a plasticizer at 750 µL/g chitosan [69]. The fruits of the *Prunus domestica* L. (plum) cultivar ‘Angeleno’, before treatment, were firstly surface-sterilized with sodium hypochlorite (0.5%) and then rinsed with SDW. The disinfected fruits were allowed to dry, and randomly divided into nine groups treated with: (i) acetic acid (1%); (ii) acetic acid (1%)+ glycerol (750 µL/g); (iii) acetic acid (1%)+ *M. laxa*; (iv) Cs.Pe; (v) Cs.Pe + *M. laxa*; (vi) C.Cs; (vii) C.Cs + *M. laxa*; (viii) C − ve (SDW); and (ix) C + ve (fruits infected with *M. laxa*). The treatment was carried out by spraying the fruits singularly with each prepared solution followed by a drying period of 2 h at room temperature (Figure S7). 

#### 4.8.2. Quality Parameters Evaluation

The quality parameters (weight loss, firmness, color change, and sugar content) were measured to evaluate the effects of the two types of chitosan on the fruits, compared to C.Cs.

(I) Weight loss

The fruit weight loss (%) for every treatment was measured at T0, T2, and T7 days after treatment (DAT) at room temperature using Equation (6) [70].

Weight loss % = (A – B)/(A × 100)(6)

where: A indicates the fruit weight at the time (0), and B indicates the fruit weight after 2 or 7 days.

(II) Peel color change

The eventual change in fruit color was measured on two sides of each treated fruit using a Colorimeter Color Analyzer (Minolta CR 400 ChromaMeter, Minolta Corp., Tokyo, Japan) at T0, T2, and T7 DAT, following Equation (7) [71].

(7) $$\Delta E=\sqrt{(\Delta L{)}^{2}+(\Delta a{)}^{2}+(\Delta b{)}^{2}}$$

where ΔE: the overall changes in color indices: L index refers to black-to-white color; a index refers to green-to-red color; b index refers blue-to-yellow color.

(III) Firmness

The firmness of the treated fruits was measured using a Fruit Pressure Tester (model FT 327, Alfonsine, Italy) with a penetrating cylinder (Ø = 8 mm). The cylinder was inserted to a constant depth of 3–5 mm into the fruit pulp at a steady speed of 2 mm per second. Firmness was determined after T7 DAT on 3 fruits per treatment, and expressed in Newtons (N), which is equivalent to kg/s^2^, compared to the negative control [72].

(IV) Sugar content

The soluble sugars in the treated fruits, primarily glucose, fructose, and sucrose, were measured using refractometry (HI96800, Hanna, Villafranca Padovana, Italy) T7 DAT on 3 fruits per treatment [73]. The values were recorded in degrees Brix, which is equivalent to 1 g sucrose/100 g liquid.

#### 4.8.3. Artificial Fungal Infection and Decay Evaluation

For the fungal infection, *M. laxa* isolated from plum, previously identified based on morphological features and molecular analysis, was used.

The sequences of the studied isolate were deposited in the NCBI GenBank with accession numbers HF678387. The studied isolate was conserved in the fungal collection of DAFE, University of Basilicata, Potenza, (Italy). A fungal suspension (10^7^ spores/mL) was prepared for fruit inoculation. After 48 h of treatment, a sterile needle was used to puncture the fruits, and 10 µL of the fungal suspension was applied to the wounded area [74]. The fruits were subsequently kept moist at room temperature in polyethylene plastic bags. The decay percentage of brown-rot disease was calculated using Equation (8), as reported by Wang et al. [75], with minor modification after 5 days of inoculation following the infection scale, as specified below. The experiment was carried out twice, with three replicates each time.

(8) $$D\mathrm{ecay}\left(\%\right)=100\times \sum (0\times N0+1\times N1+2\times N2)/(2\times N)$$

where: N0 = no decay; N1 = slight decay (≤25% of surface decay); N2 = moderate-to-high decay (more than 25% of surface decay); N = total number of fruits.

### 4.9. Statistical Analysis

The obtained results of the bioassays have been statistically analyzed by applying the ANOVA test to assess overall differences among group means using the Statistical Package for the Social Sciences (SPSS), version 13.0 (Prentice Hall: Chicago, IL, USA, 2004). After that, to identify specific differences between groups, a post hoc test was performed to evaluate the significance level, with a probability of *p* < 0.05.

## 5. Conclusions

The characteristics of chitosan extracted from *P. eryngii* contribute significantly to its potent antimicrobial activity. The presence of amino and hydroxyl groups in chitosan’s polymer structure enables it to penetrate microbial cell membranes, disrupting their integrity and leading to cell death. Moreover, studies have shown that chitosan effectively inhibits the growth of *M. laxa*, a highly damaging fungal pathogen affecting stone fruits. The antimicrobial efficacy of chitosan has sparked interest in developing formulations for diverse applications in agriculture, medicine, and industry. The findings of the current research are expected to gain more attention in the future, as fungal-derived chitosan presents a promising natural alternative for controlling serious phytopathogens, especially post-harvest, when few chemical pesticides are permitted for use in commercial formulations, either as plant-growth promoters or for crop protection.

## Figures and Tables

**Table 1:** Biomass production in three tested nutrient media, dry weight and yield % of chitin and chitosan from *P. eryngii*.

		Fungal Biomass					
**Nutrient Media**		**F.Wt (g)**	**D.Wt (g)**				
PDBW		65.5 ± 4.5	31.6 ± 2.8				
MEB		12.6 ± 2.5	4.4 ± 0.9				
NB		22.3 ± 2.2	9.4 ± 1.1				
**Extracted Ct and deacetylated Cs from biomass produced in PDBW**							
**Sample**	**pH**	**D.Wt (g)**	**Yield (%)**	**Sample**	**pH**	**D.Wt (g)**	**Yield (%)**
U.Ct.Pe	6.8	13.3 ± 1.6 _a_	42.1 ± 0.7 _a_	U.Cs.Pe	7.0	6.4 ± 0.8 _a_	48.1 ± 0.8 _ab_
B.Ct.Pe	6.9	2.6 ± 0.5 _b_	8.2 ± 0.4 _b_	B.Cs.Pe	7.0	1.9 ± 0.3 _ab_	73.1 ± 1.8 _a_

The yield percentage of chitin was calculated based on the biomass dry weight, while the yield percentage of chitosan was calculated based on the chitin dry weight. All values are expressed as mean values of 3 replicates (±SDs). Values in each column followed by different letters in lowercase are significantly different *p* < 0.05 using Tukey B test, where: (F.Wt) fresh weight; (D.Wt) dry weight; (PDBW) potato dextrose broth supplemented with 50 g of wheat powder; (MEB) malt extract broth; (NB) nutrient broth; (U.Ct.Pe) unbleached chitin; (B.Ct.Pe) bleached chitin; (U.Cs.Pe) unbleached chitosan; (B.Cs.Pe) bleached chitosan.

**Table 2:** The FT-IR bands of studied chitosan compared to commercial chitosan and chitin.

Functional Groups and Vibration Modes	B.Cs.Pe	U.Cs.Pe	C.Cs	Ct
O-H stretching of OH group	3448 vs,br	3465 s,br	3445 s,br	3463 s,br
C-H stretching of CH_2_OH group	2887 vw 2921 vw 2853 vw	2888 vw	2881 w	2886 w
ʋ (C=O) in NHCOCH_3_ group (amide I band)	1637 s	1643 s	1660 vs	1656 vs
ʋ (NH_2_) in NHCOCH_3_ group (amide II band)	1570 s	1558 m	1573 m	1560 m
δ(CH_2_) in CH_2_OH group	1426 s	1416 s	1422 m	1420 m
δ_s_(CH_3_) in NHCOCH_3_ group	1382 m	1385 s	1382 w	1374 s
ʋ_s_(C–O–C) (glycosidic linkage)	1151 w 1150 m	1152 w	1155 w	1151 s
C-N stretching	1070 vw	1071 w	1075 w	1075 w
C-C stretching	1021 w	1021 w	1025 w	1023 m
NH_2_ wagging	865 m 870 w	870 m	901 m	875 m
O-H out-of-plane bending	589 m	630 w	668 w	632 m
			[28]	

Keys: s = strong, w = weak, m = medium, br = broad, vs = very strong, vw = very weak, ν = stretching, δ = bending, cm^−1^ is wave number, where: (B.Cs.Pe) bleached chitosan; (U.Cs.Pe) unbleached chitosan; (C.Cs) commercial chitosan; (Ct) chitin.

**Table 3:** The MIC values of the studied chitosan against tested bacteria.

Samples	MIC (mg/mL) of Chitosan against Tested Bacteria			
	*E. coli*	*X. campestris*	*C. michiganensis*	*P. fluorescence*
U.Cs.Pe	1.5 ± 0.0 _a_	1.5 ± 0.0 _a_	0.375 ± 0.0 _a_	3.0 ± 0.0 _a_
B.Cs.Pe	1.5 ± 0.0 _a_	3.0 ± 0.0 _b_	0.75 ± 0.0 _b_	3.0 ± 0.0 _a_
C.Cs	3.0 ± 0.0 _b_	3.0 ± 0.0 _b_	1.5 ± 0.0 _c_	3.0 ± 0.0 _a_

All values are expressed as mean values of 3 replicates (± SDs). Values in each column followed by different letters are significantly different at *p* < 0.05 using the Tukey B test. Where: (MIC) minimum inhibitory concentration; (U.Cs.Pe) unbleached chitosan; (B.Cs.Pe) bleached chitosan; (C.Cs) commercial chitosan; (*E. coli*) *Escherichia coli*; (*X. campestris*); (*C. michiganensis*) *Clavibacter michiganensis*; (*P. fluorescence*) *Pseudomonas fluorescence*.

**Table 4:** MBC values of the studied chitosan against tested bacteria.

	MBC (mg/mL) of Chitosan against Tested Bacteria			
Samples	*E. coli*	*X. campestris*	*C. michiganensis*	*P. fluorescence*
U.Cs.Pe	6.0 ± 0.0 _a_	3.0 ± 0.0 _a_	0.75 ± 0.0 _a_	3.0 ± 0.0 _a_
B.Cs.Pe	6.0 ± 0.0 _a_	6.0 ± 0.0 _b_	1.5 ± 0.0 _b_	>6.0 ± 0.0 _b_
C.Cs	6.0 ± 0.0 _a_	6.0 ± 0.0 _b_	3.0 ± 0.0 _c_	>6.0 ± 0.0 _b_

All values are expressed as mean values of 3 replicates (± SDs). Values in each column followed by different letters are significantly different at *p* < 0.05 using the Tukey B test. Where: (MBC) minimum bactericidal concentration; (U.Cs.Pe) unbleached chitosan; (B.Cs.Pe) bleached chitosan; (C.Cs) commercial chitosan.

**Table 5:** The MIC values of the studied chitosan against tested fungi.

	MIC (mg/mL) of Chitosan against Tested Fungi		
Samples	*P. expansum*	*B. cinerea*	*M. laxa*
U.Cs.Pe	1.5 ± 0.0 _a_	0.375 ± 0.0 _a_	0.75 ± 0.0 _a_
B.Cs.Pe	3.0 ± 0.0 _b_	1.5 ± 0.0 _b_	1.5 ± 0.0 _b_
C.Cs	1.5 ± 0.0 _a_	1.5 ± 0.0 _b_	1.5 ± 0.0 _b_

All values are expressed as mean values of 3 replicates (±SDs). Values in each column followed by different letters are significantly different at *p* < 0.05 using the Tukey B test. Where: (MIC) minimum inhibitory concentration; (U.Cs.Pe) unbleached chitosan; (B.Cs.Pe) bleached chitosan; (C.Cs) commercial chitosan; (*P. expansum*) *Penicillium expansum*; (*B. cinerea*) *Botrytis cinerea*; (*M. laxa*) *Monilinia laxa*.

**Table 6:** MFC values of the studied chitosan against tested fungi.

	MFC (mg/mL) of Chitosan against Tested Fungi		
Samples	*P. expansum*	*B. cinerea*	*M. laxa*
U.Cs.Pe	6.0 ± 0.0 _a_	3.0 ± 0.0 _a_	0.75 ± 0.0 _a_
B.Cs.Pe	6.0 ± 0.0 _a_	6.0 ± 0.0 _b_	1.5 ± 0.0 _b_
C.Cs	6.0 ± 0.0 _a_	6.0 ± 0.0 _b_	1.5 ± 0.0 _b_

All values are expressed as mean values of 3 replicates (±SDs). Values in each column followed by different letters are significantly different at *p* < 0.05 using the Tukey B test. Where: (MFC) minimum fungicidal concentration; (U.Cs.Pe) unbleached chitosan; (B.Cs.Pe) bleached chitosan; (C.Cs) commercial chitosan.

**Table 7:** Phytotoxic effect of studied chitosan on *L. sativum*, *L. sativa*, and *S. lycopersicum*.

Plant	Studied Chitosan	PPM	S.G. (%)	R.E. (cm)	G.I. (%)
*L. sativum*	B.Cs.Pe	3000	20 ± 0.6	0.24 ± 0.1	0.3 ± 0.1 d
		1500	30 ± 1.5	0.53 ± 0.1	3.1 ± 1.5 c
		750	70 ± 1.0	2.57 ± 1.0	21.6 ± 5.6 b
	U.Cs.Pe	3000	10 ± 0.6	0.20 ± 0.1	0.2 ± 0.1 d
		1500	20 ± 0.6	0.42 ± 0.1	2.7 ± 0.7 c
		750	60 ± 1.0	1.23 ± 0.7	16.7 ± 2.3 b
	C.Cs	3000	30 ± 0.6	2.41 ± 0.4	8.4 ± 2.3 bc
		1500	40 ± 1.7	5.01 ± 1.1	24.1 ± 8.0 b
		750	80 ± 1.5	7.73 ± 0.6	53.4 ± 10.2 a
	C − ve (H_2_O)		87 ± 2.5	9.4 ± 1.2	75.1 ± 9.2 a
*L. sativa*	B.Cs.Pe	3000	0 ± 0	0 ± 0	0 ± 0 d
		1500	0 ± 0	0 ± 0	0 ± 0 d
		750	50 ± 0.6	0.5 ± 0.1	2.8 ± 0.7 c
	U.Cs.Pe	3000	0 ± 0	0 ± 0	0 ± 0 d
		1500	50 ± 1.2	3.2 ± 1.4	20.4 ± 8.1 b
		750	80 ± 1.2	4.1 ± 1.6	50.6 ± 8.9 a
	C.Cs	3000	0 ± 0	0 ± 0	0 ± 0 d
		1500	30 ± 0.6	0.4 ± 0.1	1.9 ± 0.5 c
		750	80 ± 1.5	1.0 ± 0.2	10.1 ± 4.6 b
	C − ve (H_2_O)		88 ± 1.7	7.5 ± 1.8	68.1 ± 7.2 a
*S. lycopersicum*	B.Cs.Pe	3000	50 ± 0.6	0.5 ± 0.1	2.3 ± 0.4 d
		1500	70 ± 0.5	4.5 ± 0.1	40.4 ± 2.2 b
		750	100 ± 0.8	7.1 ± 0.2	63.5 ± 8.5 a
	U.Cs.Pe	3000	50 ± 0.4	2.1 ± 0.1	9.8 ± 3.2 cd
		1500	70 ± 0.5	3.5 ± 0.2	27.1 ± 2.4 c
		750	90 ± 0.7	5.9 ± 0.3	54.4 ± 4.6 a
	C.Cs	3000	30 ± 0.6	0.4 ± 0.1	1.3 ± 0.9 d
		1500	50 ± 0.5	2.2 ± 0.1	10.4 ± 2.3 cd
		750	60 ± 0.5	3.8 ± 0.2	24.8 ± 3.5 c
	C − ve (H_2_O)		92 ± 5.4	12 ± 1.2	72.4 ± 5.3 a

Where: (B.Cs.Pe) bleached chitosan; (U.Cs.Pe) unbleached chitosan; (C.Cs) commercial chitosan; (C − ve) negative control (H_2_O); (PPM) the tested concentration in parts per million; (S.G. %) seed germination percentage; (R.E. cm) radical elongation measured in centimetres; (G.I. %) germination index percentage. Values of G.I. (%) followed by different letters are significantly different at *p* < 0.05 using Tukey B test.

**Table 8:** Mitotic index, normal and abnormal phase indices, total abnormalities in non-dividing and dividing cells after treating *Vicia faba* root tips with the studied chitosan, Et = Exposure time (24 h).

Treatments	% MI	Phase Index (PI)								% Total Abnormal (Tab)	
		% Prophase		% Metaphase		% Anaphase		% Telophase		Interphase	Mitosis
		Mitotic	Abn.	Mitotic	Abn.	Mitotic	Abn.	Mitotic	Abn.		
B.Cs.Pe	16.87 ± 1.01 *	14.76	0.00	45.87	9.34	21.34	5.76	18.03	1.98	0.00 ± 0.00	17.08 ± 1.23 ns
U.Cs.Pe	16.92 ± 1.03 *	13.88	0.76	40.23	8.45	23.87	7.34	22.02	2.1	0.00 ± 0.00	18.65 ± 1.98 ns
C.Cs	12.67 ± 0.97 *	15.98	3.87	57.87	23.67	13.98	7.45	12.17	3.54	1.87 ± 0.05	38.53 ± 2.77 ns
C − ve (H_2_O)	14.87 ± 1.86	16.98	2.34	55.34	14.09	18.87	6.23	8.81	1.98	0.87 ± 0.02	24.64 ± 2.98

Where: (B.Cs.Pe) bleached chitosan; (U.Cs.Pe) unbleached chitosan; (C.Cs) commercial chitosan; (C − ve) negative control (H_2_O); (% MI) mitotic index; (PI) phase index; (Tab) total abnormalities (Tab); (*) values are significant and (ns) values are not significant.

## Data Availability

Data available on request from the authors.

## Supplementary Materials

The following supporting information can be downloaded at: https://www.mdpi.com/article/10.3390/plants13182598/s1, Figure S1: deacetylation reaction of chitin to produce chitosan; Figure S2: MICs of antibacterial activity; Figure S3: MICs of antifungal activity; Figure S4: the effect of the studied chitosan on peel color of plums; Figure S5: fruits treated with the studied chitosan and infected with *M. laxa*; Figure S6: schematic diagram of the preparation of the chromosomal aberrations slide of treated *Vicia faba* root tips.; Figure S7: schematic diagram of in vivo trial.
