# Supplementary material for: Biochemical Characterization and Disease Control Efficacy of Pleurotus eryngii-Derived Chitosan—An In Vivo Study against Monilinia laxa, the Causal Agent of Plum Brown Rot
Source: Plants (Basel). 2024 Sep 17;13(18):2598. doi: 10.3390/plants13182598 (PMC11435330; doi:10.3390/plants13182598)
Supplement: Supplementary file 1 [file plants-13-02598-s001.zip › plants-3147970-supplementary.pdf]

Supplementary Materials

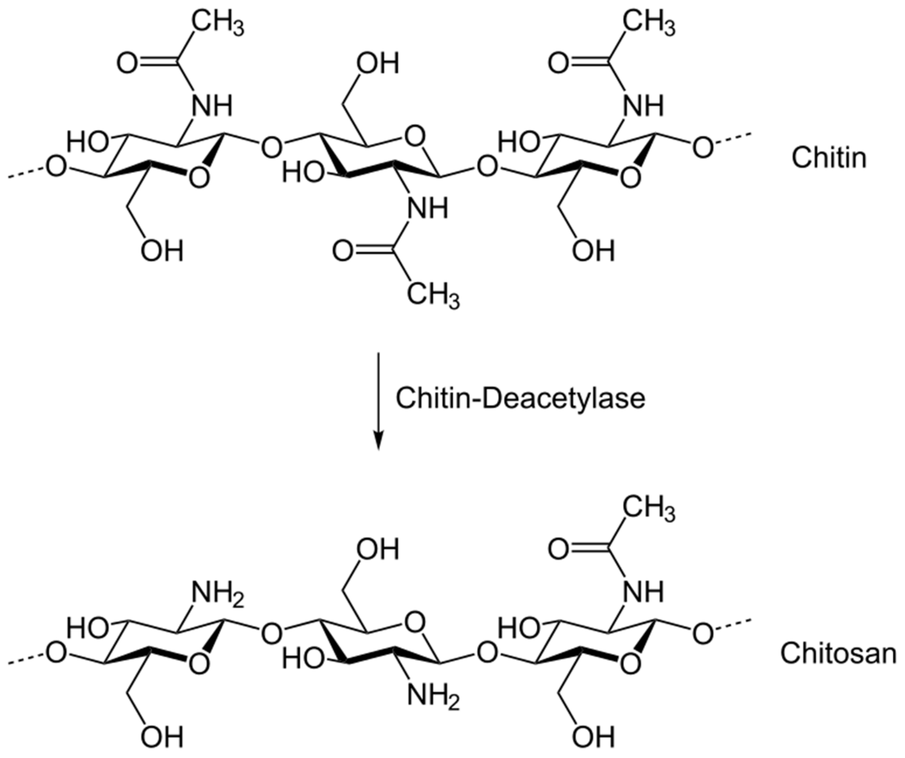

Figure S1. Deacetylation reaction of chitin to produce chitosan

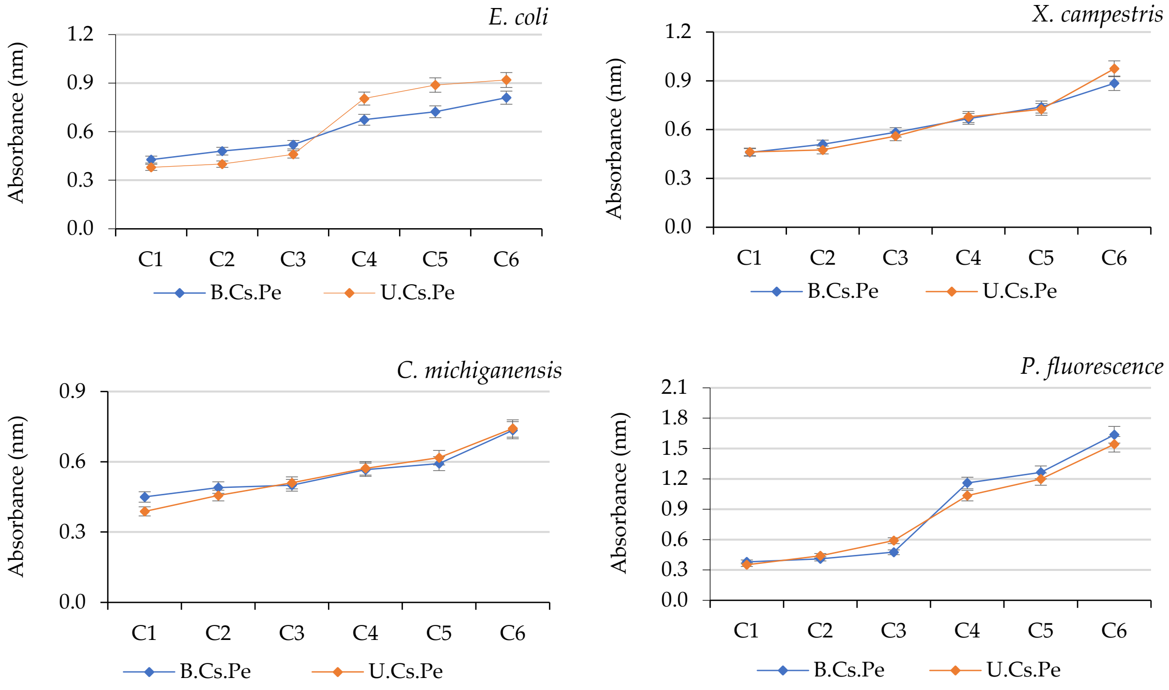

Figure S2. MICs of antibacterial activity

Where: B.Cs.Pe, bleached chitosan; U.Cs.Pe, unbleached chitosan. All values are expressed as mean values of 3 replicates (± SDs). The tested concentrations, labeled C1 to C6 are 6.0, 3.0, 1.5, 0.75, 0.375, and 0.187 mg/mL.

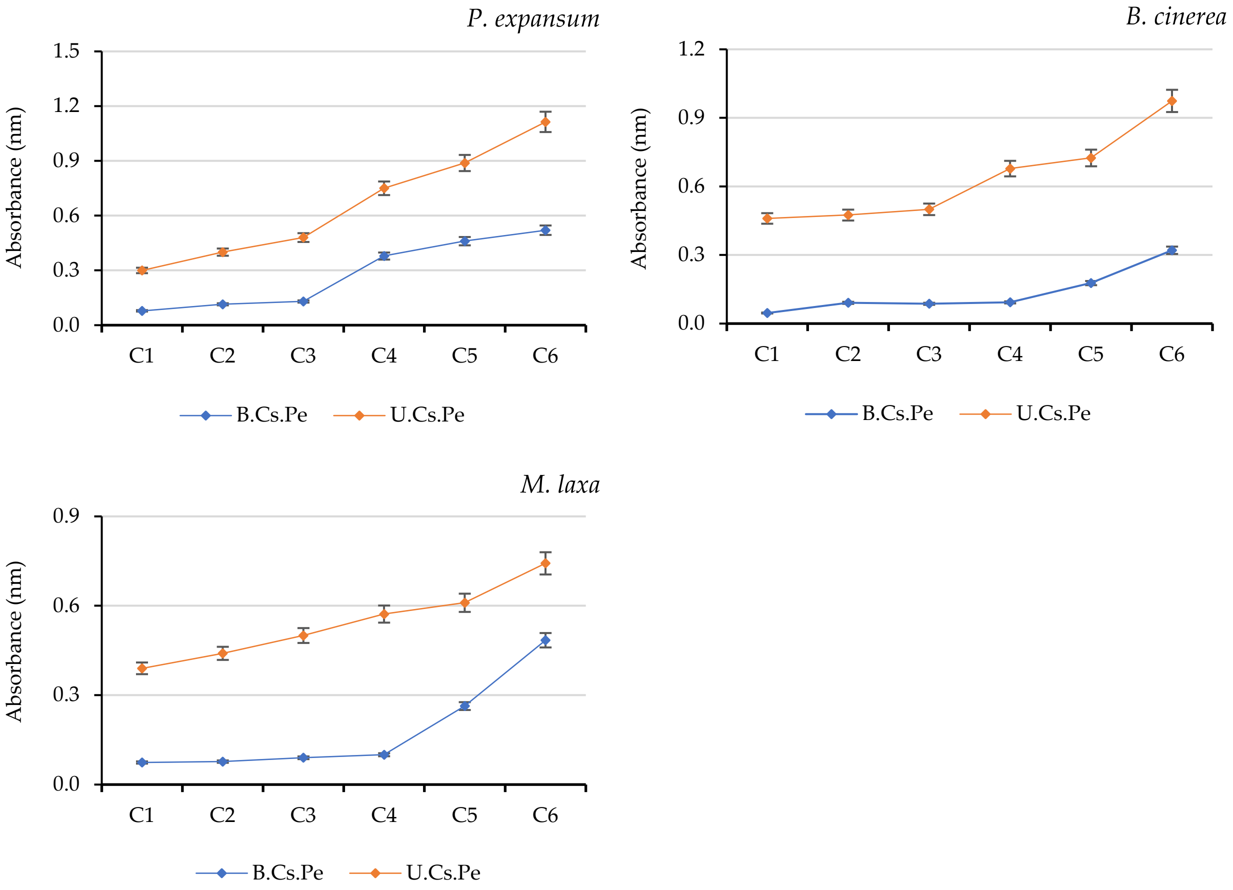

Figure S3. MICs of antifungal activity.
All values are expressed as mean values of 3 replicates (± SDs).

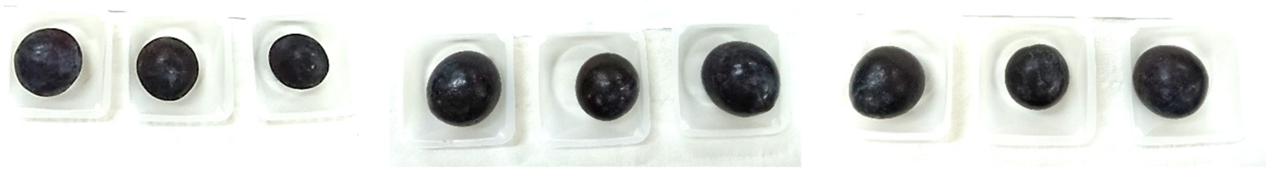

(A) **(B)** **(C)**

Figure S4. The effect of the studied chitosan on peel colour of plum fruits.
Where: (A) C-ve fruits treated only with water; (B) fruits treated with C.Cs; and (C) treated fruits with Cs-Pe.

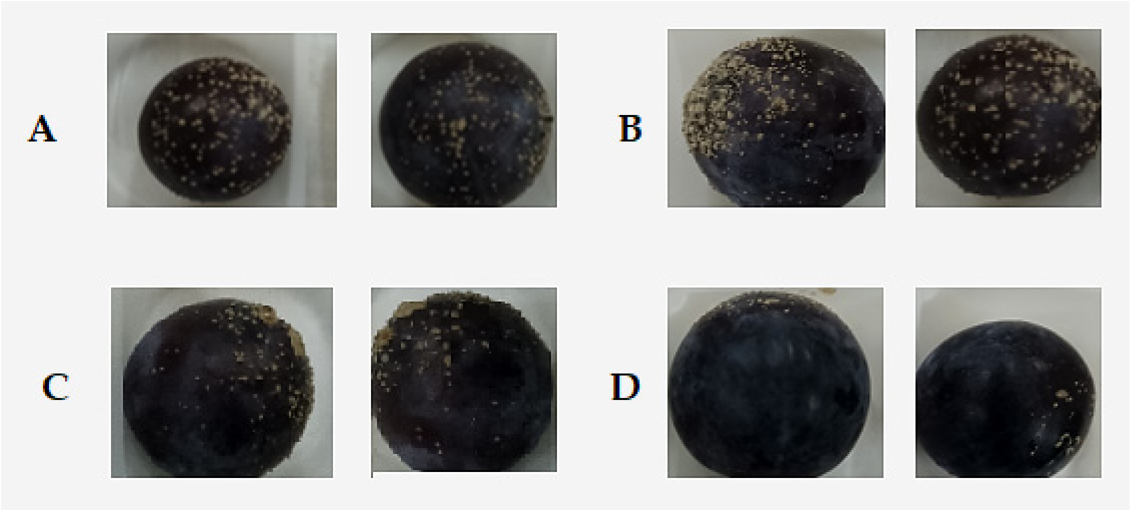

Figure S5. Fruits treated with the studied chitosan and infected with *M. laxa*.
Where: (A) C+ve fruits infected only with *M. laxa*; (B) fruits treated with acetic acid (1%) and infected with *M. laxa*; (C) treated fruits with C.Cs and infected with *M. laxa*; (D) treated fruits with Cs.Pe and infected with *M. laxa*.

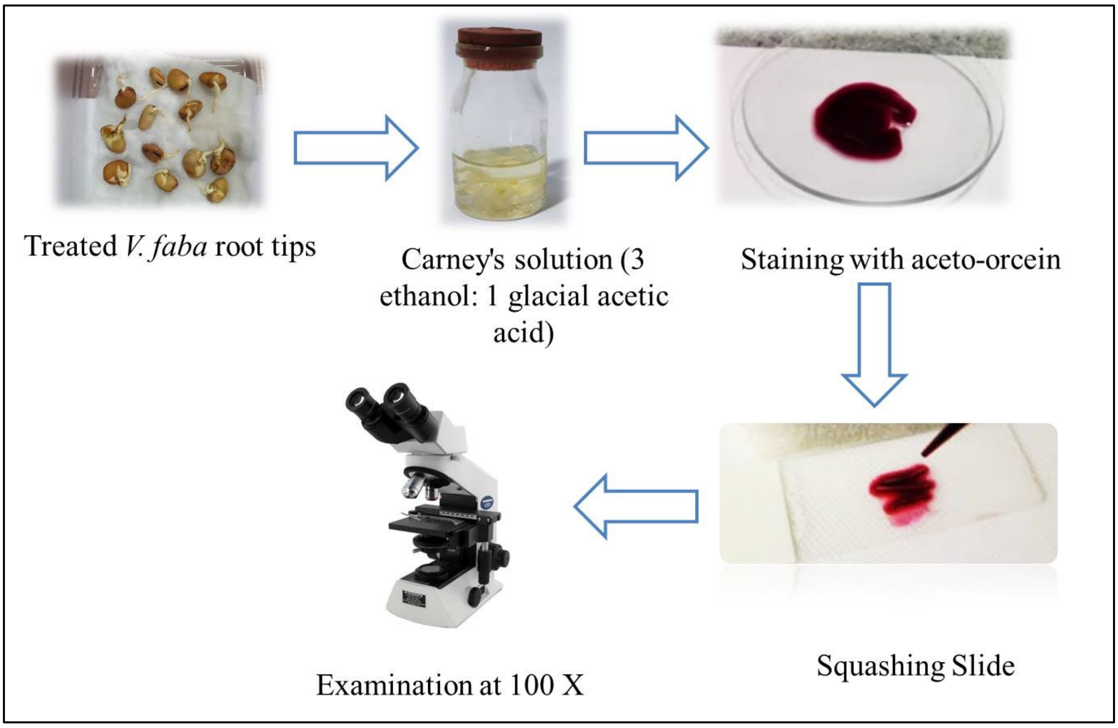

Figure S6. Schematic diagram of preparation the chromosomal aberrations slide of treated *V. faba* root tips.

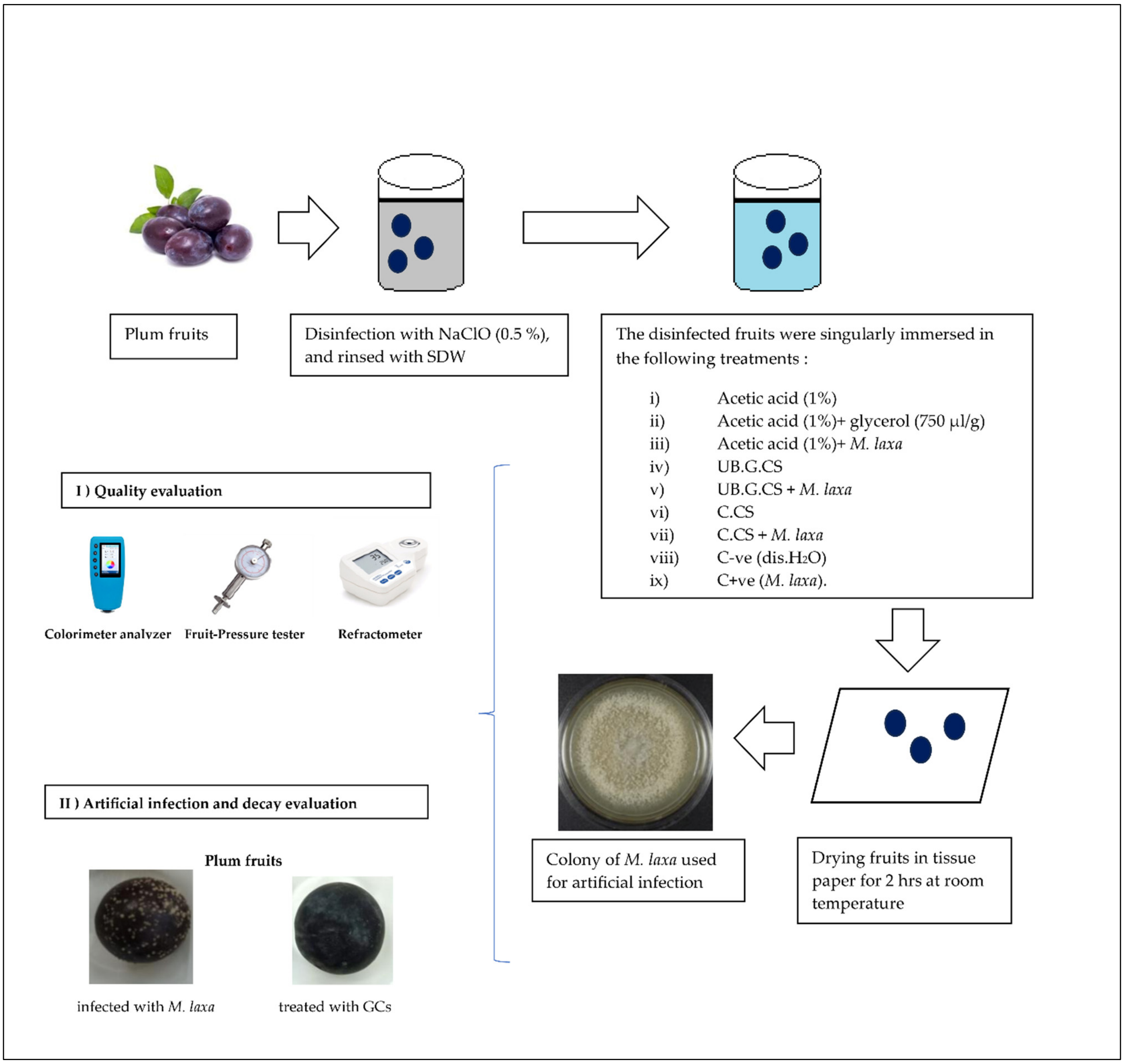

Figure S7. Schematic diagram of *in vivo* trial
